# Bacteriocin‐phage interaction (BaPI): Phage predation of *Lactococcus* in the presence of bacteriocins

**DOI:** 10.1002/mbo3.1308

**Published:** 2022-08-08

**Authors:** Claudia Rendueles, Susana Escobedo, Ana Rodríguez, Beatriz Martínez

**Affiliations:** ^1^ Department Technology and Biotechnology of Dairy Products Instituto de Productos Lácteos de Asturias (IPLA), CSIC Villaviciosa Asturias Spain

**Keywords:** bacteriocin, bacteriophage, dairy starters, *Lactococcus*, milk fermentation

## Abstract

Bacteriophages infecting dairy starter bacteria are a leading cause of milk fermentation failure and strategies to reduce the risk of phage infection in dairy settings are demanded. Along with dairy starters, bacteriocin producers (protective cultures) or the direct addition of bacteriocins as biopreservatives may be applied in food to extend shelf‐life. In this work, we have studied the progress of infection of *Lactococcus cremoris* MG1363 by the phage sk1, in the presence of three bacteriocins with different modes of action: nisin, lactococcin A (LcnA), and lactococcin 972 (Lcn972). We aimed to reveal putative bacteriocin‐phage interactions (BaPI) that could be detrimental and increase the risk of fermentation failure due to phages. Based on infections in broth and solid media, a synergistic effect was observed with Lcn972. This positive sk1‐Lcn972 interaction could be correlated with an increased burst size. sk1‐Lcn972 BaPI occurred independently of a functional SOS and cell envelope stress response but was lost in the absence of the major autolysin AcmA. Furthermore, BaPI was not exclusive to the sk1‐Lcn972 pairing and could be observed with other phages and lactococcal strains. Therefore, bacteriocins may facilitate phage predation of dairy lactococci and their use should be carefully evaluated.

## Graphical abstract

In this study, we have studied how bacteriocins may influence the progress of phage infection in dairy starter bacteria. Our results show that phage predation is indeed facilitated by particular bacteriocins depending on their mode of action, anticipating detrimental consequences for milk fermentations.

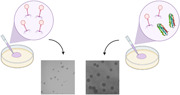

## 1 INTRODUCTION

Food fermentation can be regarded as one of the most ancient biotechnological applications of microorganisms worldwide, not only to extend the shelf‐life of perishable food but to produce palatable, nutritious, and healthy products (Shiferaw Terefe & Augustin, 2020). Currently, food fermentations are driven by tailored starter cultures to ensure product quality and batch‐to‐batch reproducibility. However, the prevailing conditions in food fermentations (e.g., large volumes and high‐density cultures of one or a few selected starter strains) are very favorable for the proliferation of bacteriophages, viruses that exclusively propagate in bacteria. Cheese manufacture is highly susceptible to phage contamination because milk is not sterile and pasteurization is not always effective enough to inactivate phages infecting the dairy starter *Lactococcus lactis* (Madera et al., 2004; Wagner et al., 2017). In the presence of infecting phages, milk acidification slows down resulting in low‐quality end‐products or even in a complete fermentation failure and loss of the whole batch which is translated into severe economic losses. Therefore, strategies to reduce the risk of phage infection in dairy settings are still demanded (Mahony & van Sinderen, 2015; Pujato et al., 2019; Romero et al., 2020). Conversely, bacteriophages infecting pathogenic bacteria may play a desirable role in food fermentations as tools for controlling the presence of unwanted bacteria (Fernandez et al., 2017; Gonçalves de Melo et al., 2018).

In the context of food safety, the use of bacteriocins and bacteriocin producers as biopreservatives and protective cultures, respectively, has gained attention as means to extend shelf‐life, while diminishing the presence of pathogenic bacteria (O'Connor et al., 2020; Silva et al., 2018). Bacteriocins are ribosomally‐synthesized antimicrobial peptides that can inhibit target bacteria by disrupting cell envelope functions, either by pore formation, inhibition of cell wall biosynthesis, cell wall degradation or by hitting intracellular targets (Pérez‐Ramos et al., 2021; Telhig et al., 2020). Along with the rapid production of lactic acid, bacteriocins are behind the ability of lactic acid bacteria to displace competitors and thrive in nutritious environments such as milk.

We have recently reviewed the outcomes of the combined use of bacteriocins and bacteriophages in food biopreservation (Rendueles et al., 2022). Synergistic or additive effects are often reported, mirroring what is currently known for antibiotic and phage combinations. PAS or phage‐antibiotic synergy (Comeau et al., 2007) is a phenomenon by which subinhibitory concentrations of certain antibiotics stimulate phage production. Self‐evidently, PAS has been studied in the clinical context and focused on clinically relevant pathogenic bacteria. Mechanistic insights revealed the complexity of PAS, where synergism, additivism, or antagonism may be observed, even with the same antibiotic depending on the stoichiometry, the mode of action of the antibiotic, the presence of antibiotic resistance mechanisms, the phage species, and the environmental conditions (Gu Liu et al., 2022). Enhanced phage production is frequently achieved with antibiotics inhibiting cell wall biosynthesis, associated with increases in both plaque size and phage burst size and/or delayed lysis (Comeau et al., 2007; Gu Liu et al., 2022; Kim et al., 2018). Interestingly, antibiotics may also synergize with temperate phages by specifically selecting against lysogens (Al‐Anany et al., 2021).

Prompted by these reports, we have realized that knowledge of plausible bacteriocin‐phage interactions (BaPIs) in starter lactic acid bacteria is scarce. In this study, we asked the question of whether bacteriocins, which can be naturally produced or intentionally added to fermented products, interfere positively or negatively with phages infecting dairy *Lactococcus*. In other words, how risky, in terms of fermentation failure, the use of bacteriocins and protective cultures is in the likely scenario of phage contamination. To this end, we have studied the fate of *Lactococcus cremoris* MG1363 infected with the *Skunavirus* sk1 in the presence of nisin, lactococcin A (LcnA), and lactococcin 972 (Lcn972), three bacteriocins with different modes of action. An insight into the underlying mechanisms behind BaPI was also approached.

## 2 MATERIALS AND METHODS

### 2.1 Bacterial strains, culturing conditions, and bacteriophage propagation

*L. cremoris* MG1363 (Gasson, 1983) was routinely grown in M17 (Formedium) supplemented with 0.5% glucose (GM17) at 30°C without agitation. Other lactococcal strains and mutants used in this work are described in Table 2.

To propagate sk1 bacteriophage (Pillidge & Jarvis, 1988), *L. cremoris* MG1363 was grown in GM17 with 10 mM Ca(NO_3_)_2_ and 10 mM MgSO_4_ (GM17**) until an optical density at 600 nm (OD_600_) of 0.5 and infected with sk1 at a multiplicity of infection (MOI) of 0.03. The lysate was centrifugated at 3400*g* for 30 min at 8°C, filtered through a polyethersulfone filter (0.2 µm), and stored at 4°C. Phage enumeration (pfu ml^−1^) was performed by the double‐layer agar assay in GM17**, inoculating the top agar (0.7%) with 100 µl of an overnight culture of *L. cremoris* MG1363 or any other lactococcal host and appropriate lysate dilutions prepared in SM buffer (20 mM Tris HCl, pH 7.5. 100 mM NaCl, 10 mM Ca(NO_3_)_2_, 10 mM MgSO_4_). Other phages used in this work are described in Table 2.

### 2.2 Bacteriocin preparations

Nisin was dissolved in 0.05% (v/v) acetic acid at 100 µg ml^−1^ and Lcn972 at 25,600 AU ml^−1^ in 50 mM sodium phosphate buffer, pH 6.8 (NaPi). LcnA at 50 AU ml^−1^ was prepared from the cell‐free supernatant of *L. lactis* B190, a LcnA producer (Diep et al., 2007), grown in GM17 at 30°C for 8 h. Stock solutions were stored at −20°C.

#### 2.2.1 sk1 infection in the presence of bacteriocins

Bacterial growth was followed in a microtiter plate reader (Tecan Trading AG). Ninety‐six‐well plates were prepared with different final concentrations of sk1 phage (10^6^, 10^4^, and 10^2^pfu ml^−1^) and Lcn972 (5, 2.5, and 1.25 AU ml^−1^), nisin (30, 15, and 7.5 ng ml^−1^) or LcnA (3, 1.5 and 0.75 AU ml^−1^). Wells were inoculated with exponentially growing *L. cremoris* MG1363 at 5 × 10^7^ CFU ml^−1^ (final concentration), and the OD_600_ was measured every 15 min for 24 h. Control cultures with only sk1, only bacteriocins, and without any treatment (positive control) were also included. The experiments were repeated twice.

To analyze the results, the area under the curve (AUC) of the treated cultures and the positive control for the first 5 h of incubation was measured as previously described (Xie et al., 2018). The percentage of inhibition (PI) was calculated by normalizing the AUC of treated cultures by that of the positive control (1‐AUC_treated_/AUC_control_ × 100). Synergism or positive BaPI was defined when the sum of the PI of each component alone was lower than the PI of the combination (PI_only bacteriocin_ + PI_only phage_)/PI_bacteriocin + phage_ < 1.

### 2.3 Plaque size

Infection by sk1 and other phages in the presence of bacteriocins was also studied by the double‐layer agar assay with bacteriocins added to the upper layer (Lcn972: 5 and 10 AU ml^−1^; nisin: 20, 30, and 40 ng ml^−1^; LcnA: 5, 2.5, and 1.25 AU ml^−1^), along with the appropriate lactococcal host. The diameter of the plaques was measured using a digital caliper. Increments over 30% compared to the diameter of the plaque size on the reference lactococcal strain were regarded as relevant, based on the variability of the measurements.

### 2.4 Phage adsorption

Phage adsorption was performed as described (Madera et al., 2003). Phage sk1 was added at an MOI of 0.001 to *L. cremoris* MG1363 cells diluted to an OD_600_ of 0.8 in GM17** with Lcn972 at a final concentration of 5 AU ml^−1^ or the same volume of NaPi buffer for 15 min. Samples were removed every 5 min. After centrifugation, the nonabsorbed phages (residual) were quantified. A sample without cells was equally treated to determine the initial phage titer. Experiments were carried out with three biological replicates. The percentage of adsorption was calculated as (1 − residual phage titer/initial phage titer) × 100.

### 2.5 One‐step growth curves

One‐step growth curves were conducted as described (Moineau et al., 1993) with some modifications. *L. cremoris* cells were grown in GM17 and collected at OD_600_ of 0.8. Cells (2 ml) were resuspended in 900 μl of GM17** and phage sk1 was added at an MOI of 0.1. After adsorption for 5 min and washing to remove free phages, cells were diluted up to 10^−4^ in GM17** (control) or GM17** supplemented with Lcn972 at 5 and 1.25 AU ml^−1^ and incubated at 30°C. Samples were withdrawn every 5 min for 60 min, and subsequent dilutions were assayed by the double‐layer agar method to determine the burst size (pfu per infected cell) and latent period, determined by approximation as the midpoint of the exponential phase. Each one‐step growth curve was performed three times.

### 2.6 Statistical analyses

Comparisons were assessed by a one‐tailed *t*‐test as implemented in Microsoft Excel 2019 (Microsoft Corporation). *p* < 0.05 was considered to be significant.

## 3 RESULTS

### 3.1 Synergistic and neutral BaPI depending on the bacteriocin mode of action

**Figure 1:**
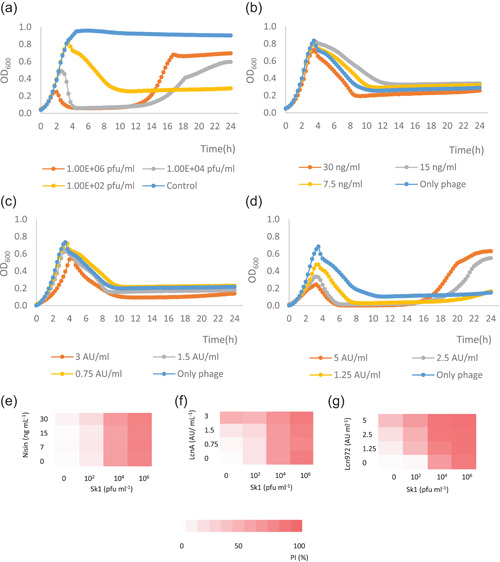
Bacteriocin‐phage interactions. Representative growth curves of *Lactococcus cremoris* MG1363 infected with phage sk1 (a) and with sk1 10^2^ pfu ml^−1^ and nisin (b), LcnA (c), or Lcn972 (d). Heatmaps of the percentage of inhibition (PI) within the first 5 h of incubation with sk1, bacteriocins, or both for nisin (e), LcnA (f), or Lcn972 (g).

**Figure A1:**
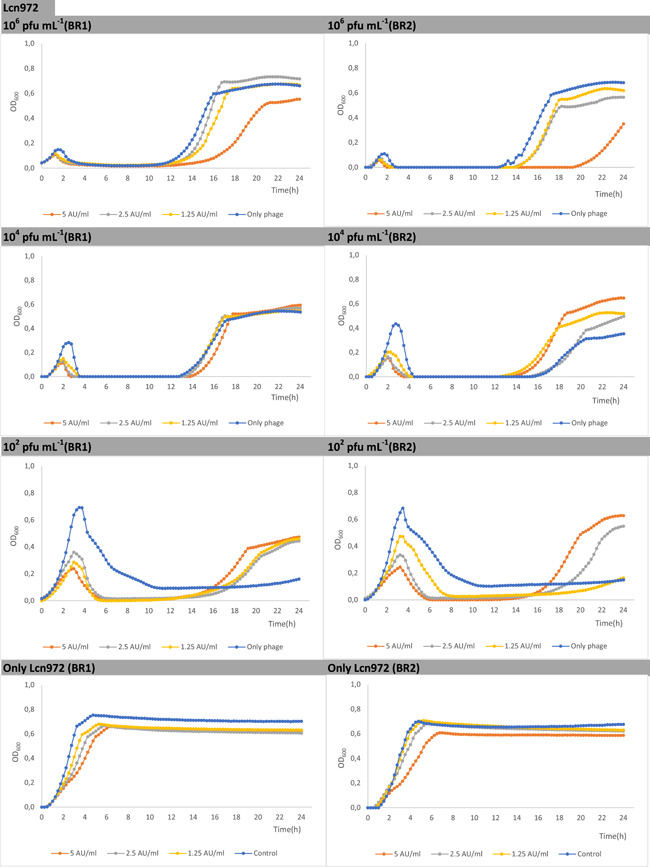
Growth curves of *Lactococcus cremoris* MG1363 infected with the phage sk1 (10^6^, 10^4^, 10^2^, or 0 pfu ml^−1^) and different concentrations of Lcn972. Two biological replicates (BR1 and BR2) are shown.

**Figure A2:**
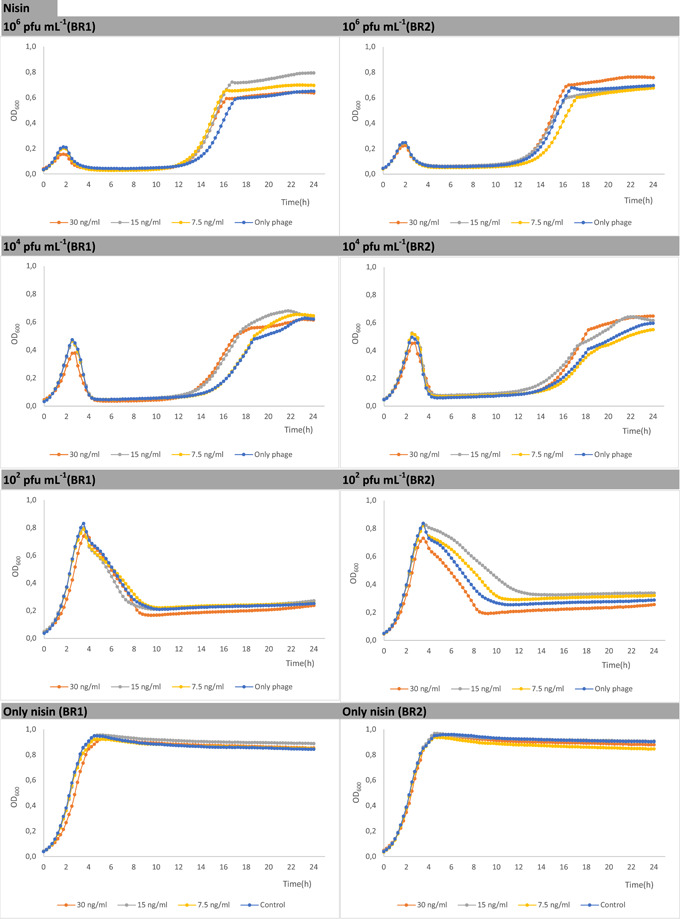
Growth curves of *Lactococcus cremoris* MG1363 infected with the phage sk1 (10^6^, 10^4^, 10^2^, or 0 pfu ml^−1^) and different concentrations of nisin. Two biological replicates (BR1 and BR2) are shown.

**Figure A3:**
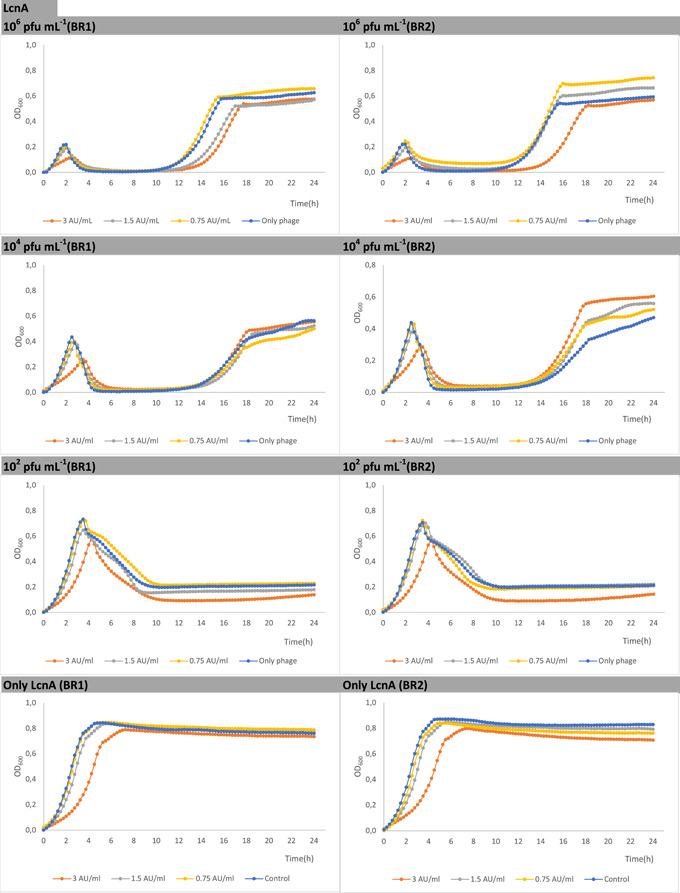
Growth curves of *Lactococcus cremoris* MG1363 infected with the phage sk1 (10^6^, 10^4^, 10^2^, or 0 pfu ml^−1^) and different concentrations of LcnA. Two biological replicates (BR1 and BR2) are shown.

Three bacteriocins with antimicrobial activity against *L. cremoris* MG1363 were chosen to reveal putative interactions during infection by phage sk1: the lantibiotic nisin and the two nonmodified bacteriocins LcnA and Lcn972. Nisin, which is currently authorized as a food preservative worldwide, combines pore formation with binding of the cell wall precursor lipid II, thereby inhibiting cell wall biosynthesis as well (Wiedemann et al., 2001). LcnA binds to the mannose PTS transporter and kills lactococci by pore formation (Diep et al., 2007). Lcn972 binds to lipid II and inhibits cell wall biosynthesis precisely at the division septum (Martínez et al., 2000, 2008). Preliminary experiments were initially carried out to select for those bacteriocin concentrations that did not or marginally inhibit growth (Figures A1, A2, A3). Phage infection was studied in broth following the OD_600_ of an exponentially‐growing *L. cremoris* MG1363 infected by sk1 (Figure 1), using a range of concentrations of both sk1 and each bacteriocin. The growth curves of infected *L. cremoris* MG1363 without bacteriocins showed that the onset of lysis was slightly delayed and occurred at higher cell densities as the initial MOI decreased (Figure 1a).

Representative growth curves of *L. cremoris* MG1363 infected with sk1 10^2^ pfu ml^−1^ (MOI 2 × 10^−6^) in the presence of bacteriocins are shown in Figure 1b–d. All the growth curves are depicted in the appendices (Figures A1, A2, A3). Lcn972 had a major impact on the progress of infection (Figure 1d). To compare the growth curves in the different conditions, the AUC was calculated and the PI, referred to as an untreated culture, was visualized as a heatmap (Figure 1e–g). Inhibition of *L. cremoris* MG1363 growth with nisin and LcnA was comparable to that of the phage‐only control (Figure 1e,f), whereas there was a significant synergism with Lcn972, that is, a stronger inhibition than the sum of each treatment alone (Figure 1g). The positive interaction (*p* < 0.05) occurred with Lcn972 at 5 and 2.5 AU ml^−1^ in the cultures infected with 10^2^ pfu ml^−1^. In the combinations with Lcn972 and sk1 at 10^−4^ pfu ml^−1^ (MOI 2 × 10^−4^), lysis also occurred at a lower OD_600_ alongside increasing Lcn972 concentrations (Figure A1). At the highest level of initial phage infection, growth curves again resemble that of the phage‐only control and a similar PI was recorded regardless of the bacteriocin (Figure 1g).

### 3.2 The emergence of phage resistance but no bacteriocin resistance

At high phage sk1 concentrations (10^4^ and 10^6^ pfu ml^−1^), growth of *L. cremoris* MG1363 resumed, presumably due to the emergence of phage‐resistant mutants. In some instances, Lcn972‐treated cultures recovered earlier, even when their counterparts with the phage sk1 alone (10^2^ pfu ml^−1^) did not (Figure 1d). To determine if cross‐resistance might have occurred, the resistant phenotype of the cells in the wells with regrowth was assessed. Colonies (*n* = 16) were isolated after 24 h of incubation from the combinations of sk1 (10^2^ pfu ml^−1^) with Lcn972 at 5 and 2.5 AU ml^−1^. For comparison, colonies were also isolated from those treated with sk1 alone (10^6^ pfu ml^−1^). Bacterial lawns were tested for phage and Lcn972 resistance by spotting sk1 (10^7^, 10^6^, and 10^5^ pfu ml^−1^) and Lcn972 (400, 200, 100 AU ml^−1^). All the surviving cells were resistant to sk1 but remained as susceptible to Lcn972 as the parental *L. cremoris* MG1363.

### 3.3 Enlarged phage plaques are formed in the presence of bacteriocins

**Figure 2:**
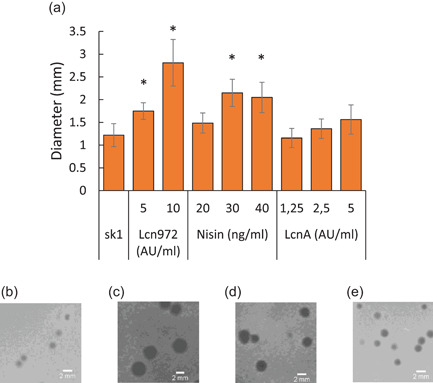
Plaque diameter of phage sk1 in the presence of Lcn972, nisin, and LcnA (a). Plaque size in GM17 plates without bacteriocin (b) and with Lcn972 10 AU ml^−1^ (c), nisin 30 ng ml^−1^ (d), or LcnA 2.5 AU ml^−1^ (e).

Phage infection in the presence of bacteriocins was also tested in solid media in double‐layer agar assays by adding the bacteriocins to the soft agar and the diameter of sk1 plaques was measured after overnight incubation. In agreement with the infections in broth, the average sk1 plaque size increased over 30% when Lcn972 (5 AU ml^−1^) was added and was enlarged up to 130% with 10 AU ml^−1^ (Figure 2). In the case of nisin, while with the lowest concentration (20 ng ml^−1^) the increment was below 30%, higher concentrations (30 and 40 ng ml^−1^) resulted in a 75% increase, reaching a maximum with 30 ng ml^−1^ (Figure 2). By contrast, no increase was triggered by LcnA at any of the tested concentrations.

### 3.4 The increased burst size is likely behind the sk1‐Lcn972 synergistic BaPI

**Figure A4:**
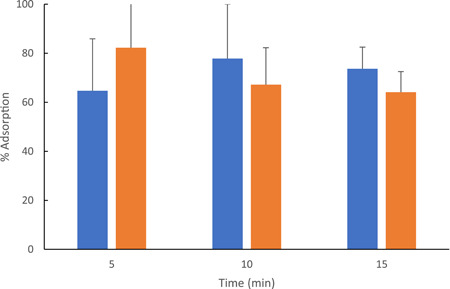
Rate of adsorbed sk1 phage at different times of incubation with a multiplicity of infection of 0.001 in GM17** (blue) or GM17** with Lcn972 5 AU ml^−1^ (orange).

Due to the significant impact of Lcn972 on sk1 infection, further experiments were carried out to characterize this event. Because Lcn972 is a cationic peptide and binds to the cell envelope, we determined if Lcn972 at 5 AU ml^−1^, a concentration at which a positive interaction with sk1 was observed, facilitated adsorption of the phage sk1 to the cells. Similar values of adsorbed phages were measured regardless of the bacteriocin with 67.2 ± 8.4 percentage of adsorbed phages in GM17** supplemented with Lcn972 at 5 AU ml^−1^ versus 77.8 ± 8.8 without it after 10 min (Figure A4).

Next, the progress of infection in the presence of Lcn972 was evaluated by one‐step growth curves. Exponentially growing cells were infected at an MOI of 0.1 and, after adsorption for 5 min, cells were diluted and resuspended in GM17** with Lcn972 at 5 and 1.25 AU ml^−1^. While the latent period did not change compared to the control without bacteriocin, the burst size increased significantly (*p* < 0.05) by 43.6% and 39.8% proportionally to Lcn972 concentrations, respectively (Table 1).

**Table 1:** The burst size and latent period based on one‐step curves of sk1 infecting *L. lactis* MG1363 in the absence or presence of Lcn972.

Condition	Burst size (pfu ml^−1^)	Latent period (min)
Control	43 ± 4	27 ± 3
Lcn972 (1.25 AU ml^−1^)	60* ± 10	28 ± 2
Lcn972 (5 AU ml^−1^)	61** ± 1	26 ± 2

*Note*: Values are the mean and standard deviation of three biological replicates.

Significantly different means compared to the control (one‐tail Student *t‐*test). **p < 0.01; *p < 0.05.

### 3.5 sk1‐Lcn972 BaPI in several genetic backgrounds

Taking plaque size as a proxy for positive BaPIs, the interaction between sk1 and Lcn972 was studied in different genetic backgrounds (Table 2) to get an insight into host factors that may be involved. Increments in plaque size were observed in a mutant devoid of *recA* showing that positive BaPI occurs independently of the SOS response. Likewise, positive BaPi still happens in the absence of the resident prophage TP712, pointing to a minor role of prophages in this event. Two mutants either overexpressing or lacking *cesSR* were also tested because Lcn972 is known to trigger the cell envelope stress response through the two‐component system (TCS) CesSR (Martínez et al., 2007). Moreover, this TCS also responds to phage infection (Fallico et al., 2011). Although the increments in plaque size were small, larger plaques were observed with 10 AU ml^−1^ Lcn972, regardless of the *cesSR* mutation. Hence, a functional cell envelope stress response seems not to be behind sk1‐Lcn972 BaPI. Of note, BaPi was lost in a mutant lacking the major autolysin AcmA (Table 2), anticipating a main role of the host autolytic machinery in this event.

**Table 2:** Phage plaque size and increments in the presence of the bacteriocin Lcn972

			Ø mm				
			Lcn972 AU ml^‐1^			Increment (%)	
Phage	Host	Characteristics (reference)	0 (*n*)	5 (*n*)	10 (*n*)	5	10
sk1	MG1363	Wild type (Gasson, 1983)	1.22 ± 0.25 (120)	1.75 ± 0.18 (40)	2.81 ± 0.51 (40)	42.9	129.8
	Δ*recA*	Lacks RecA (Duwat et al., 1995)	1.31 ± 0.24 (80)	1.75 ± 0.23 (40)	3.22 ± 0.33 (40)	36.1	155.7
	ΔTP712	Lacks the resident prophage TP712 (Escobedo et al., 2021)	1.53 ± 0.24 (78)	2.17 ± 0.29 (40)	2.84 ± 0.29 840)	41.7	85.4
	Δ*acmA*	Lacks the major autolysin AcmA (Buist et al., 1995)	1.47 ± 0.29 (80)	1.86 ± 0.28 (20)	1.87 ± 0.24 (20)	26.6	27.5
	NZ9000 Δ*cesSR*	Lacks the two‐component system CesSR (Pinto et al., 2011)	1.62 ± 0.22 (80)	1.87 ± 0.28 (40)	2.25 ± 0.37 (40)	15.4	38.7
	NZ9000 pIL252::*cesSR*	Multicopy *cesSR* (Pinto et al., 2011)	1.56 ± 0.31 (80)	1.71 ± 0.25 (40)	2.24 ± 0.31 (40)	10	44
P2	MG1363	*Skunavirus* (Moineau et al., 1995)	1.31 ± 0.20 (40)	1.88 ± 0.34 (20)	2.56 ± 0.33 (20)	43.01	94.79
CHPC1183	MG1363	*Ceduovirus* (López‐González et al., 2018)	1.74 ± 0.64 (60)	1.61 ± 0.49 (25)	2.44 ± 0.83 (10)	−7.9	39.8
c2	MG1363	*Ceduovirus* (Pillidge & Jarvis, 1988)	1.78 ± 0.32 (70)	1.94 ± 0.32 (40)	2.11 ± 0.46 (40)	8.7	18.4
bIL170	IL1403	*Skunavirus* (Crutz‐Le Coq et al., 2002)	1.33 ± 0.26 (60)	2.43 ± 0.39 (30)	ND	83.7	ND

Abbreviation: *n*, number of measured plaques.

### 3.6 Positive BaPI is not exclusive to the sk1‐Lcn972 pair

In a similar fashion as above, plaque size enlargement was assessed with other available phages and lactococcal strains in the presence of Lcn972 (Table 2). Positive BaPI was observed with the phage p2, which is closely related to sk1 (93% of identity at the amino acid level). It was also observed with the C*eduovirus* CHPC1183, but not with c2. Nevertheless, the increment in plaque size was small and detected only with Lcn972 at 10 AU ml^−1^ (Table 2). Positive BaPI was evident with phage bIL170, in this case infecting the lactococcal strain *L. lactis* IL1403.

## 4 DISCUSSION

The results reported in this study demonstrate that phage predation of dairy lactococci may be favored in the presence of bacteriocins. Although this idea has not been systematically examined so far, the result was somewhat expected. In previous reports, bacteriocins had been shown to act synergistically with phages infecting foodborne pathogenic bacteria, as reviewed by Rendueles et al. (2022). Moreover, enhanced production of phages infecting lactococci with other antimicrobial agents (e.g., penicillin) or peptidoglycan destabilizing compounds (e.g., glycine) has been empirically used in the past to facilitate visualization of pinpoint plaques (Lillehaug, 1997; Mahony, Tremblay, et al., 2015).

Based on the results from both phage infection in broth and enlargement of phage plaques in the presence of the three bacteriocins with a different mode of action, inhibition of cell wall biosynthesis appears to be driving BaPI in lactococci. This is exemplified by the pairing of Lcn972 and sk1 that showed the stronger positive BaPI, followed by nisin, and the lack of interactions with the pore‐forming LcnA. Therefore, like in the case of PAS, antibiotic activity may determine the type of interaction (Gu Liu et al., 2022; Liu et al., 2022). Nevertheless, many other extrinsic and intrinsic factors have been correlated with enhanced phage production by subinhibitory concentrations of antibiotics. In this work, for example, the bacteriocins and the phage sk1 were added simultaneously but stronger PAS can be attained by altering the time and sequence of each treatment (Akturk et al., 2019; Chaudhry et al., 2017). Therefore, BaPIs may take place in other scenarios that those studied here.

The positive BaPI between sk1 and Lcn972 could be correlated with an increased burst size, while other intrinsic phage properties involved in PAS such as a higher adsorption rate or an extended latent period (Liu et al., 2022) did not occur. The increased burst size might be explained by the particular mode of action of Lcn972. On one hand, contrary to pore‐forming bacteriocins that stop immediately all energy‐driven reactions in the bacterial cell, Lcn972 inhibits cell division only and the cells remain metabolically active for approximately 45 min, that is, for one division cycle (Martínez et al., 2000). This scenario would allow phage replication to proceed undisturbed. On the other hand, Lcn972 treated cells swell, a structural change in (ovo‐)cocci that, similarly to filamentation in rod‐shaped bacteria, has often been related to increased phage production in the presence of antibiotics (Comeau et al., 2007; Kim et al., 2018).

It is also conceivable that a weaker cell wall due to inhibition of cell wall biosynthesis by Lcn972 might also facilitate the peptidoglycan‐degrading activity of the phage endolysin. Then, a larger fraction of infected host cells would lyse, accelerating phage spread. Indeed, this was apparent in broth infections where *L. cremoris* cultures lysed at lower ODs when Lcn972 is present (see Figure 1d). A larger progeny may also explain why sk1 BIMs developed faster in the combined sk1‐Lcn972 treatments, reflecting what happens when sk1 was added alone at high MOIs. In line with this reasoning, the absence of plaque enlargement when the sk1‐Lcn972 combination was tested on a mutant devoid of the major autolysin AcmA was an important finding. Host autolysins are required by some phages to resume their lytic cycle (Frias et al., 2009), but its involvement in PAS (or BaPI) has not been described so far and, definitively, deserves further investigation. On the contrary, positive sk1‐Lcn972 BaPi occurs independently of other host factors such as the integrity of the TCS CesSR or a functional SOS response as described for PAS (Comeau et al., 2007).

Finally, it is worth mentioning that positive BaPi is not exclusive to the sk1‐Lcn972 pairing and it could be also observed with unrelated phages (e.g., CHPC1183 and bIL170) and with other lactococcal species, despite the limited number of phage:host combinations tested in this work.

## 5 CONCLUSIONS

Considering that bacteriocin production is a widespread trait in lactococci and, in lactic acid bacteria in general, further work is warranted to fully appreciate the impact of bacteriocins *in situ*, that is, during both natural and industrial milk fermentations. Nevertheless, the results so far suggest that adding bacteriocin producers (or protective cultures) alongside starter bacteria might increase the risk of fermentation delays, due to the conceivable synergistic BaPis that may occur as reported in this work.

## AUTHOR CONTRIBUTIONS

**Claudia Rendueles**: Formal analysis (equal); investigation (lead); writing—original draft (equal); writing—review and editing (equal). **Susana Escobedo**: Investigation (equal); methodology (equal); supervision (equal); writing—review and editing (equal). **Ana Rodríguez**: Conceptualization (equal); funding acquisition (equal); project administration (equal); writing—review and editing (equal). **Beatriz Martínez**: Conceptualization (equal); formal analysis (equal); funding acquisition (equal); project administration (equal); supervision (equal); writing—original draft (lead).

## CONFLICT OF INTEREST

None declared.

## ETHICS STATEMENT

None required.

## Data Availability

All data are provided in full in the results section of this paper. Raw data are available at the institutional repository of the Spanish National Research Council, Digital.CSIC: https://doi.org/10.20350/digitalCSIC/14698

## References

[mbo31308-bib-0001] Akturk, E. , Oliveira, H. , Santos, S. B. , Costa, S. , Kuyumcu, S. , Melo, L. D. R. , & Azeredo, J. (2019). Synergistic action of phage and antibiotics: Parameters to enhance the killing efficacy against mono and dual‐species biofilms. Antibiotics (Basel), 8(3), 103. 10.3390/antibiotics8030103 PMC6783858 31349628

[mbo31308-bib-0002] Al‐Anany, A. M. , Fatima, R. , & Hynes, A. P. (2021). Temperate phage‐antibiotic synergy eradicates bacteria through depletion of lysogens. Cell Reports, 35, 109172. 34038739 10.1016/j.celrep.2021.109172

[mbo31308-bib-0003] Buist, G. , Kok, J. , Leenhouts, K. J. , Dabrowska, M. , Venema, G. , & Haandrikman, A. J. (1995). Molecular cloning and nucleotide sequence of the gene encoding the major peptidoglycan hydrolase of *Lactococcus lactis*, a muramidase needed for cell separation. Journal of Bacteriology, 177, 1554–1563. 7883712 10.1128/jb.177.6.1554-1563.1995 PMC176772

[mbo31308-bib-0004] Chaudhry, W. N. , Concepción‐Acevedo, J. , Park, T. , Andleeb, S. , Bull, J. J. , & Levin, B. R. (2017). Synergy and order effects of antibiotics and phages in killing *Pseudomonas aeruginosa* biofilms. PLoS One, 12, e0168615. 28076361 10.1371/journal.pone.0168615 PMC5226664

[mbo31308-bib-0005] Comeau, A. M. , Tétart, F. , Trojet, S. N. , Prère, M.‐F. , & Krisch, H. M. (2007). Phage‐antibiotic synergy (PAS): β‐Lactam and quinolone antibiotics stimulate virulent phage growth. PLoS One, 2, e799. 17726529 10.1371/journal.pone.0000799 PMC1949050

[mbo31308-bib-0006] Crutz‐Le Coq, A.‐M. , Cesselin, B. , Commissaire, J. , & Anba, J. (2002). Sequence analysis of the lactococcal bacteriophage bIL170: Insights into structural proteins and HNH endonucleases in dairy phages. Microbiology, 148, 985–1001. 11932445 10.1099/00221287-148-4-985

[mbo31308-bib-0007] Diep, D. B. , Skaugen, M. , Salehian, Z. , Holo, H. , & Nes, I. F. (2007). Common mechanisms of target cell recognition and immunity for class II bacteriocins. Proceedings of the National Academy of Sciences of the United States of America, 104, 2384–2389. 17284603 10.1073/pnas.0608775104 PMC1892938

[mbo31308-bib-0008] Duwat, P. , Ehrlich, S. D. , & Gruss, A. (1995). The recA gene of *Lactococcus lactis*: Characterization and involvement in oxidative and thermal stress. Molecular Microbiology, 17, 1121–1131. 8594331 10.1111/j.1365-2958.1995.mmi_17061121.x

[mbo31308-bib-0009] Escobedo, S. , Wegmann, U. , Pérez de Pipaon, M. , Campelo, A. B. , Stentz, R. , Rodríguez, A. , & Martínez, B. (2021). Resident TP712 prophage of *Lactococcus lactis* strain MG1363 provides extra holin functions to the P335 phage CAP for effective host lysis. Applied and Environmental Microbiology, 87, e0109221. 34260308 10.1128/AEM.01092-21 PMC8432518

[mbo31308-bib-0010] Fallico, V. , Ross, R. P. , Fitzgerald, G. F. , & McAuliffe, O. (2011). Genetic response to bacteriophage infection in *Lactococcus lactis* reveals a four‐strand approach involving induction of membrane stress proteins, d‐alanylation of the cell wall, maintenance of proton motive force, and energy conservation. Journal of Virology, 85, 12032–12042. 21880765 10.1128/JVI.00275-11 PMC3209278

[mbo31308-bib-0011] Fernandez, L. , Escobedo, S. , Gutierrez, D. , Portilla, S. , Martinez, B. , Garcia, P. , & Rodriguez, A. (2017). Bacteriophages in the dairy environment: From enemies to allies. Antibiotics (Basel), 6, 27. 10.3390/antibiotics6040027 PMC5745470 29117107

[mbo31308-bib-0012] Frias, M. J. , Melo‐Cristino, J. , & Ramirez, M. (2009). The autolysin LytA contributes to efficient bacteriophage progeny release in *Streptococcus pneumoniae* . Journal of Bacteriology, 191, 5428–5440. 19581370 10.1128/JB.00477-09 PMC2725628

[mbo31308-bib-0013] Gasson, M. J. (1983). Plasmid complements of *Streptococcus lactis* NCDO 712 and other lactic streptococci after protoplast‐induced curing. Journal of Bacteriology, 154, 1–9. 6403500 10.1128/jb.154.1.1-9.1983 PMC217423

[mbo31308-bib-0014] Gonçalves de Melo, A. , Levesque, S. , & Moineau, S. (2018). Phages as friends and enemies in food processing. Current Opinion in Biotechnology, 49, 185–190. 28987913 10.1016/j.copbio.2017.09.004

[mbo31308-bib-0015] Gu Liu, C. , Green, S. I. , Min, L. , Clark, J. R. , Salazar, K. C. , Terwilliger, A. L. , Kaplan, H. B. , Trautner, B. W. , Ramig, R. F. , & Maresso, A. W. (2022). Phage‐antibiotic synergy is driven by a unique combination of antibacterial mechanism of action and stoichiometry. mBio, 11, e01462‐20. 10.1128/mBio.01462-20 PMC7407087 32753497

[mbo31308-bib-0016] Kim, M. , Jo, Y. , Hwang, Y. J. , Hong, H. W. , Hong, S. S. , Park, K. , & Myung, H. (2018). Phage‐antibiotic synergy via delayed lysis. Applied and Environmental Microbiology, 84(22), e02085‐18. 30217844 10.1128/AEM.02085-18 PMC6210123

[mbo31308-bib-0017] Lillehaug, D. (1997). An improved plaque assay for poor plaque‐producing temperate lactococcal bacteriophages. Journal of Applied Microbiology, 83, 85–90. 9246774 10.1046/j.1365-2672.1997.00193.x

[mbo31308-bib-0018] Liu, C. , Hong, Q. , Chang, R. Y. , Kwok, P. C. , & Chan, H.‐K. (2022). Phage‐antibiotic therapy as a promising strategy to combat multidrug‐Resistant infections and to enhance antimicrobial efficiency. Antibiotics (USSR), 11, 570. 10.3390/antibiotics11050570 PMC9137994 35625214

[mbo31308-bib-0019] López‐González, M. J. , Escobedo, S. , Rodríguez, A. , Neves, A. R. , Janzen, T. , & Martínez, B. (2018). Adaptive evolution of industrial *Lactococcus lactis* under cell envelope stress provides phenotypic diversity. Frontiers in Microbiology, 9, 17. 30455679 10.3389/fmicb.2018.02654 PMC6230721

[mbo31308-bib-0020] Madera, C. , García, P. , Janzen, T. , Rodríguez, A. , & Suárez, J. E. (2003). Characterisation of technologically proficient wild *Lactococcus lactis* strains resistant to phage infection. International Journal of Food Microbiology, 86, 213–222. 12915032 10.1016/s0168-1605(03)00042-4

[mbo31308-bib-0021] Madera, C. , Monjardin, C. , & Suarez, J. E. (2004). Milk contamination and resistance to processing conditions determine the fate of *Lactococcus lactis* bacteriophages in dairies. Applied and Environmental Microbiology, 70, 7365–7371. 15574937 10.1128/AEM.70.12.7365-7371.2004 PMC535134

[mbo31308-bib-0022] Mahony, J. , & van Sinderen, D. (2015). Novel strategies to prevent or exploit phages in fermentations, insights from phage‐host interactions. Current Opinion in Biotechnology, 32, 8–13. 25300036 10.1016/j.copbio.2014.09.006

[mbo31308-bib-0023] Mahony, J. , Tremblay, D. M. , Labrie, S. J. , Moineau, S. , & van Sinderen, D. (2015). Investigating the requirement for calcium during lactococcal phage infection. International Journal of Food Microbiology, 201, 47–51. 25744695 10.1016/j.ijfoodmicro.2015.02.017

[mbo31308-bib-0024] Martínez, B. , Boettiger, T. , Schneider, T. , Rodríguez, A. , Sahl, H.‐G. , & Wiedemann, I. (2008). Specific interaction of the unmodified bacteriocin lactococcin 972 with the cell wall precursor lipid II. Applied and Environmental Microbiology, 74, 4666–4670. 18539790 10.1128/AEM.00092-08 PMC2519333

[mbo31308-bib-0025] Martínez, B. , Rodríguez, A. , & Suárez, J. E. (2000). Lactococcin 972, a bacteriocin that inhibits septum formation in lactococci. Microbiology, 146, 949–955. 10784053 10.1099/00221287-146-4-949

[mbo31308-bib-0026] Martínez, B. , Zomer, A. L. , Rodríguez, A. , Kok, J. , & Kuipers, O. P. (2007). Cell envelope stress induced by the bacteriocin Lcn972 is sensed by the lactococcal two‐component system CesSR. Molecular Microbiology, 64, 473–486. 17493129 10.1111/j.1365-2958.2007.05668.x

[mbo31308-bib-0027] Moineau, S. , Durmaz, E. , Pandian, S. , & Klaenhammer, T. R. (1993). Differentiation of two abortive mechanisms by using monoclonal antibodies directed toward lactococcal bacteriophage capsid proteins. Applied and Environmental Microbiology, 59, 208–212. 16348844 10.1128/aem.59.1.208-212.1993 PMC202079

[mbo31308-bib-0028] Moineau, S. , Walker, S. A. , Vedamuthu, E. R. , & Vandenbergh, P. A. (1995). Cloning and sequencing of LlaDCHI restriction/modification genes from *Lactococcus lactis* and relatedness of this system to the *Streptococcus pneumoniae* DpnII system. Applied and Environmental Microbiology, 61, 2193–2202. 7793939 10.1128/aem.61.6.2193-2202.1995 PMC167490

[mbo31308-bib-0029] O'Connor, P. M. , Kuniyoshi, T. M. , Oliveira, R. P. , Hill, C. , Ross, R. P. , & Cotter, P. D. (2020). Antimicrobials for food and feed; a bacteriocin perspective. Current Opinion in Biotechnology, 61, 160–167. 31968296 10.1016/j.copbio.2019.12.023

[mbo31308-bib-0030] Pérez‐Ramos, A. , Madi‐Moussa, D. , Coucheney, F. , & Drider, D. (2021). Current knowledge of the mode of action and immunity mechanisms of LAB‐bacteriocins. Microorganisms, 9, 2107. 34683428 10.3390/microorganisms9102107 PMC8538875

[mbo31308-bib-0031] Pillidge, C. J. , & Jarvis, A. W. (1988). DNA restriction maps and classification of the lactococcal bacteriophages c2 and skl. Journal of Dairy Science and Technology, 23, 411–416.

[mbo31308-bib-0032] Pinto, J. P. , Kuipers, O. P. , Marreddy, R. K. , Poolman, B. , & Kok, J. (2011). Efficient overproduction of membrane proteins in *Lactococcus lactis* requires the cell envelope stress sensor/regulator couple CesSR. PLoS One, 6, e21873. 21818275 10.1371/journal.pone.0021873 PMC3139573

[mbo31308-bib-0033] Pujato, S. A. , Quiberoni, A. , & Mercanti, D. J. (2019). Bacteriophages on dairy foods. Journal of Applied Microbiology, 126, 14–30. 30080952 10.1111/jam.14062

[mbo31308-bib-0034] Rendueles, C. , Duarte, A. C. , Escobedo, S. , Fernández, L. , Rodríguez, A. , García, P. , & Martínez, B. (2022). Combined use of bacteriocins and bacteriophages as food biopreservatives. A review. International Journal of Food Microbiology, 368, 109611. 35272082 10.1016/j.ijfoodmicro.2022.109611

[mbo31308-bib-0035] Romero, D. A. , Magill, D. , Millen, A. , Horvath, P. , & Fremaux, C. (2020). Dairy lactococcal and streptococcal phage–host interactions: An industrial perspective in an evolving phage landscape. FEMS Microbiology Reviews, 44, 909–932. 33016324 10.1093/femsre/fuaa048

[mbo31308-bib-0036] Shiferaw Terefe, N. , & Augustin, M. A. (2020). Fermentation for tailoring the technological and health related functionality of food products. Critical Reviews in Food Science and Nutrition, 60, 2887–2913. 31583891 10.1080/10408398.2019.1666250

[mbo31308-bib-0037] Silva, C. C. G. , Silva, S. P. M. , & Ribeiro, S. C. (2018). Application of bacteriocins and protective cultures in dairy food preservation. Frontiers in Microbiology, 9, 594. 29686652 10.3389/fmicb.2018.00594 PMC5900009

[mbo31308-bib-0038] Telhig, S. , Ben Said, L. , Zirah, S. , Fliss, I. , & Rebuffat, S. (2020). Bacteriocins to thwart bacterial resistance in gram negative bacteria. Frontiers in Microbiology, 11, 2807. 10.3389/fmicb.2020.586433 PMC7680869 33240239

[mbo31308-bib-0039] Wagner, N. , Samtlebe, M. , Franz, C. M. A. P. , Neve, H. , Heller, K. J. , Hinrichs, J. , & Atamer, Z. (2017). Dairy bacteriophages isolated from whey powder: Thermal inactivation and kinetic characterisation. International Dairy Journal, 68, 95–104.

[mbo31308-bib-0040] Wiedemann, I. , Breukink, E. , van Kraaij, C. , Kuipers, O. P. , Bierbaum, G. , de Kruijff, B. , & Sahl, H. G. (2001). Specific binding of nisin to the peptidoglycan precursor lipid II combines pore formation and inhibition of cell wall biosynthesis for potent antibiotic activity. Journal of Biological Chemistry, 276, 1772–1779. 11038353 10.1074/jbc.M006770200

[mbo31308-bib-0041] Xie, Y. , Wahab, L. , & Gill, J. J. (2018). Development and validation of a microtiter plate‐based assay for determination of bacteriophage host range and virulence. Viruses, 10, 189. 10.3390/v10040189 PMC5923483 29649135

